# The Hypothermic Influence on CHOP and Ero1-α in an Endoplasmic Reticulum Stress Model of Cerebral Ischemia

**DOI:** 10.3390/brainsci5020178

**Published:** 2015-05-15

**Authors:** Gagandip K. Poone, Henrik Hasseldam, Nina Munkholm, Rune S. Rasmussen, Nina V. Grønberg, Flemming F. Johansen

**Affiliations:** Department of Biomedical Sciences and Biotech Research & Innovation Centre (BRIC), University of Copenhagen, 2200, Denmark; E-Mails: gagandipkb@hotmail.com (G.K.P.); henrik.hasseldam@bric.ku.dk (H.H.); nmunkholm@hotmail.com (N.M.); rsr@sund.ku.dk (R.S.R.); ninavg@sund.ku.dk (N.V.G.)

**Keywords:** brain ischemia, unfolded protein response, transcription factor CHOP, Ero1-α protein, hypoxia-inducible factor-proline dioxygenases, stroke

## Abstract

Hypoxia induced endoplasmic reticulum stress causes accumulation of unfolded proteins in the endoplasmic reticulum and activates the unfolded protein response, resulting in apoptosis through CCAAT-enhancer-binding protein homologous protein (CHOP) activation. In an *in vitro* and *in vivo* model of ischemic stroke, we investigated whether hypothermia regulates the unfolded protein response of CHOP and Endoplasmic reticulum oxidoreductin-α (Ero1-α), because Ero1-α is suggested to be a downstream CHOP target. The gene expression of CHOP and Ero1-α was measured using Quantitative-PCR (Q-PCR) in rat hippocampi following global cerebral ischemia, and in hypoxic pheochromocytoma cells during normothermic (37 °C) and hypothermic (31 °C) conditions. As a result of ischemia, a significant increase in expression of CHOP and Ero1-α was observed after three, six and twelve hours of reperfusion following global ischemia. A stable increase in CHOP expression was observed throughout the time course (*p* < 0.01, *p* < 0.0001), whereas Ero1-α expression peaked at three to six hours (*p* < 0.0001). Induced hypothermia in hypoxia stressed PC12 cells resulted in a decreased expression of CHOP after three, six and twelve hours (*p* < 0.0001). On the contrary, the gene expression of Ero1-α increased as a result of hypothermia and peaked at twelve hours (*p* < 0.0001). Hypothermia attenuated the expression of CHOP, supporting that hypothermia suppress endoplasmic reticulum stress induced apoptosis in stroke. As hypothermia further induced up-regulation of Ero1-α, and since CHOP and Ero1-α showed differential regulation as a consequence of both disease (hypoxia) and treatment (hypothermia), we conclude that they are regulated independently.

## 1. Introduction

Hypothermia is a promising treatment strategy following several medical conditions such as traumatic brain injury, myocardial infarction, cardiac arrest, and ischemic stroke [1,2,3,4]. Contrary to single target therapies, hypothermia aims at multiple targets, exerting protection by altering a variety of detrimental effects. The benefits of therapeutic cooling have been demonstrated in several experimental *in vitro* and *in vivo* studies of cerebral ischemia [5,6,7,8,9]. Overall, these investigations found improvements in metabolism, inhibition of inflammatory mediators, regulation of gene expression, improved blood brain barrier (BBB) integrity, and regulation of the balance between cell death and survival following an ischemic stroke.

Cerebral ischemia causes hypoxic stress in cells, leading to endoplasmic reticulum (ER), environment disruption, and accumulation of unfolded proteins in the ER lumen. In response to cellular aggregation of misfolded and/or unfolded proteins, the Unfolded Protein Response (UPR) becomes activated [10,11]. The UPR will initially induce translational attenuation, transcriptional up-regulation of ER chaperones and enhance unfolded protein degradation in an attempt to prevent further cellular damage, overcome the insult, and restore normal ER function. If stress is severe or prolonged, the UPR triggers cell death [11,12].

In unstressed cells, the ER chaperone glucose-regulated-protein (GRP78) binds pancreatic-ER-kinase (PERK), inositol-requiring ER-transmembrane RNAse-1 (IRE-1) and activating-transcription factor-6 (ATF6), keeping them inactivated [13]. Upon stress, ER calcium depletion and, thereby, an enhanced load of unfolded proteins, GRP78 is titrated away from PERK, ATF6, and IRE-1 by binding the unfolded proteins instead, leading to activation of the UPR actors. Downstream from these pathways is the activation of the CCAAT-enhancer-binding protein homologous protein (CHOP), a pro-apoptotic transcription factor [13,14,15].

CHOP plays a central role in the ischemic damage resulting in neuronal death [10]. Many CHOP targets have been identified, amongst these growth arrest and DNA damage-inducible protein (GADD34) and Bcl-2, all serving to mediate apoptosis [16].

Endoplasmic reticulum oxidoreductin-α (Ero1-α) has also been suggested to be a CHOP target gene [15]. Ero1 exists in two forms, Ero1-α and Ero1-β. Ero1-α is an oxidoreductase that relays disulfide bonds to protein disulfide isomerase (PDI), which helps ER proteins to obtain correct conformation [15]. Some investigations also suggest that Ero1-α is responsible for inositol 1,4,5-triphosphate (IP3) receptor induced Ca^2+^ release, initiating apoptosis [16]. Thus, the association between CHOP and Ero1-α in ischemic brain damage and a possible therapeutic influence of hypothermia remains unclear. 

In an experimental animal model of global ischemia, we investigated relative changes in the expression of CHOP and Ero1-α following ischemia. Subsequently, in an *in vitro* model, we mimicked the ischemic condition by introducing hypoxia into a neuron-like cell line and applied hypothermic conditions to evaluate the effects of hypothermia on the transcriptional levels of these mediators.

## 2. Methods

### 2.1. Animals and Cell Lines

Sixty male Wistar rats (aged 9 weeks, weighing 280–300 g) obtained from Taconic (Ry, Denmark) were cared for according to the guidelines and approvals of the Department of Experimental Medicine, University of Copenhagen, Denmark, and all the experiments were conducted according to the Danish Animal Experiments Committee (#2012-DY-2934). Animals were acclimatized for 7 days prior to surgery and allowed free access to food and water under diurnal lighting conditions. All efforts were made to diminish pain, suffering and stress of the animals.

PC12 cells are a commercially available rat pheocromocytoma cell line originating from rat adrenal medulla (ATCC, Boras, Sweden). PC12 cells were grown on collagen (Sigma-Aldrich, Copenhagen, Denmark) coated (6 ug/mL) dishes with DMEM (Life Technologies, Naerum, Denmark), supplemented with horse serum (Life technologies), fetal bovine serum (Life technologies) and penicillin/streptomycin (Life technologies) (10/5/1%) and cultured in a standard incubation chamber (5% CO_2_/21% O_2_). Cells were plated at a density of 1–2 × 10^4^ cells/cm^2^.

### 2.2. Global Cerebral Ischemia

Rats were subjected to experimental global ischemia by the two-vessel occlusion (2-VO) model during systemic hypotension [17]. Anesthesia was induced (4%) and maintained (1%–2%) with isoflurane in 30%/70% NO/O_2_, and a femoral catheter was embedded to keep animals hypotensive, which also allowed for arterial blood-sampling throughout surgery. Blood gases were analyzed on a Radiometer ABL 555 blood gas analyzer (Radiometer, Brønshøj, Denmark). As global brain ischemia may impede respiratory system functions, ventilator-controlled respiration was performed with a Harvard 683 small animal ventilator (Scandidact, Odder, Denmark). Both common carotid arteries were gently exposed and systemic hypotension (50 mmHg) was induced with a heparinized syringe through the femoral catheter. Arteries were then fully ligated for 12 min. with subsequent removal of ligatures and reperfusion of 3, 6 and 12 h (*n* = 10/group). Incision wounds were treated with lidocain gel (10 mg/mL; Region Hovedstadens Apotek, Copenhagen, Denmark). At the end of reperfusion, rats were deeply anesthetized, decapitated, and brains were rapidly removed, snap frozen in isopentane and stored at −80 °C. Dorsal and ventral parts of the hippocampus were carefully dissected on ice and added TRIzol reagent (Invitrogen) to avoid RNA degradation prior to RNA isolation.

### 2.3. Thapsigargin and Hypoxia Stressed PC12 Cells

Confluent PC12 cells were incubated with 0.32 × 10^−5^ M thapsigargin (Sigma-Aldrich) for 1 h, 4 h or 24 h at either 37 °C or 31 °C in a standard incubator, in order to establish a positive control for UPR activation in the cell line. To identify suitable working concentrations, a survival MTT (Methylthiazol Tetrazolium Bromid, Sigma-Aldrich) assay was performed with 10 different thapsigargin concentrations (0.02 × 10^−5^ M to 10.24 × 10^−5^ M). A control sample of untreated PC12 cells with the lowest corresponding DMSO concentration (0.031%) was incubated for 24 h at 37 °C. One biological sample for each treatment parameter was generated.

Three biological samples of PC12 cells (grown to confluency at 37 °C) were incubated at 37 °C or 31 °C for 1 h, 3 h, 6 h or 12 h in a hypoxic incubation chamber with 0.3% O_2_, controlled with nitrogen.

Three biological control samples were incubated for 12 h in a standard incubator (5% CO_2_) at 37 °C. Biological triplicates were generated for each treatment parameter (time/temperature). At the end of incubation time, cells quickly proceeded to RNA isolation.

### 2.4. RNA Extraction and Complementary DNA (cDNA) Synthesis

RNA extraction was performed according to the TRIzol reagent protocol (Life Technologies) and measured on a BioPhotometer 6131 spectrophotometer (Eppendorf, Hamburg, Germany). 

RNA samples were reverse transcribed using the ImProm-II Reverse Transcription System (Qiagen, Copenhagen, Denmark). Thermal cycling conditions were set to 5 min at 25 °C for annealing, 60 min at 42 °C for extension and 15 min at 70 °C for inactivating the reverse transcriptase enzyme. From this reaction, a cDNA concentration of 0.05 μg/μL was obtained.

### 2.5. Quantitative-PCR

All samples were prepared in triplicates for the targets CHOP, Ero1-α, and Hmbs (normalization target). Primers for the housekeeping gene Hmbs, HPRT, synaptophysin, and β-actin were tested for stability during hypoxia under normo- and hypothermic conditions. Hmbs exhibited highly stable expression, which is why this gene was chosen as the normalization target for further studies.

The Q-PCR reactions were performed using the ABI 7300 Detection System (Life Technologies). In a 25 μL reaction, 2 μL cDNA (100 ng/2 μL), 3 μL nuclease free H_2_O, 7.5 μL relevant primers (forward + reverse, 2000 nM), and 12.5 μL RealQ-PCR dUTP Master Mixes Kit (Ampliqon, Odense, Denmark) was used for a total of 25 μL. Thermal cycling conditions were: fifteen minutes at 95 °C (for hot start of the Taq DNA polymerase enzyme), forty cycles at 95 °C for 30 s (dissociation of cDNA strings), 60 °C for 1 min (annealing of primers), and 72 °C for 30 s (extension). 

Dissociation curves were included in all runs—one cycle of 95 °C for 30 s, 50 °C for 30 s, and from 50 °C to 95 °C, with a reading every 0.5 °C and a hold for 10 s.

Primers used: Hmbs (76 base pairs (bp), F: TCTAGATGGCTCAGATAGCATGCA, R: TGGACCATCTTCTTGCTGAACA), CHOP (100 bp, F: CCT GAA AGC AGA AAC CGG TC, R: CCT CAT ACC AGG CTT CCA GC) and Ero1-α (204 bp, F: TTA AGT CTG CGA GCT ACA AGT ATT C, R: AGT AAA TCC ACA TAC TCA GCA).

### 2.6. Statistical Analysis

All data are calculated according to the comparative Ct method and expressed as mean ± SD, presenting data as fold change in gene expression normalized to housekeeping gene (Hmbs) and relative to the untreated control, an approach determining the relative quantification rather than absolute quantification [18]. Ordinary one-way analysis of variance (ANOVA) was used for comparisons between different groups followed by Tukey’s multiple comparisons test. Data indicated that temperature has an influence on the mean fold changes of both CHOP and Ero1-α during the 12 h of reperfusion. Thus, a two-way ANOVA was performed to test if there was an interaction between temperature and time followed by multiple pairwise comparisons by the Holm-Sidak method. *p* < 0.05 was considered statistically significant.

## 3. Results

### 3.1. Global Cerebral Ischemia Induces Up-regulation of CHOP and Ero1-α in Rat Hippocampi

**Figure 1:**
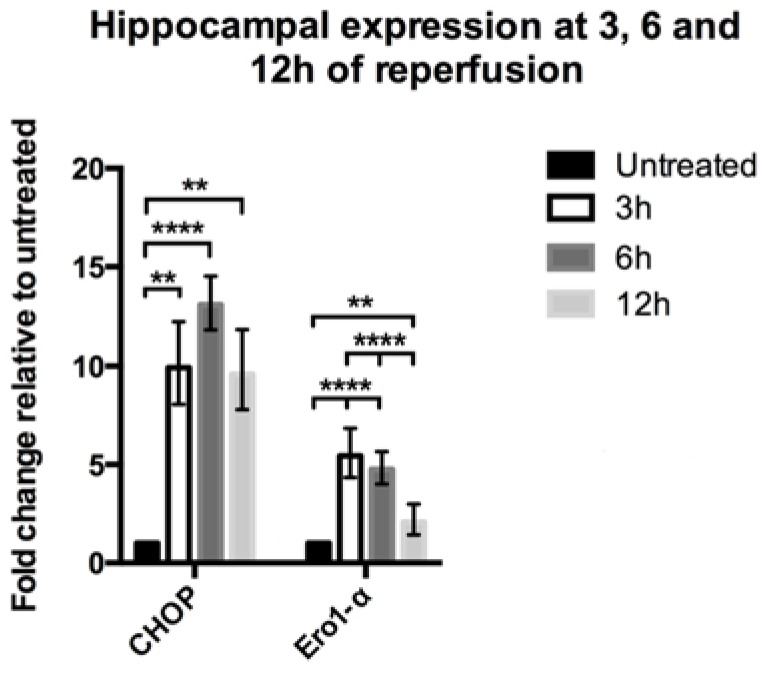
Hippocampal expression of CHOP and Ero1-α following global ischemia. Quantitative PCR revealed a significant increase in expression of CHOP and Ero1-α induced by global ischemia over a time course of 12 h. The greatest expression level of CHOP is detected at 6 h of reperfusion (*n* = 6) compared to untreated animals (*n* = 10), (*p* < 0.0001). At 3 h (*n* = 7) and 12 h (*n* = 6) after induction of ischemia similar expression levels of CHOP were observed, significantly greater than in the untreated group (*p* < 0.01). No difference between the different time points was detected. Ero1-α was significantly increased at 3 h (*n* = 5), 6 h (*n* = 7) and 12 h (*n* = 5) of reperfusion (*p* < 0.0001 and 0.01 respectively), with greatest expression levels at 3 to 6 h followed by a decrease. Data analyzed by one-way ANOVA followed by Tukey’s multiple comparisons test. ** *p* < 0.01 and **** *p* < 0.0001.

Induction of global ischemia altered the expression level of CHOP and Ero1-α in hippocampi of Wistar rats (Figure 1). A stable CHOP increase was observed throughout the entire time course. At 3 and 12 h of reperfusion, CHOP was found to be significantly up-regulated compared to untreated, control animals (*p* < 0.01). At 6 h of reperfusion, CHOP levels were further increased (*p* < 0.0001), however not significantly different from the 3 and 12 h group. Similar to CHOP, Ero1-α was also increased at 3 to 6 h following ischemia (*p* < 0.0001), although it seemed to reach maximum at this time point and was reduced towards normal levels after 12 h of reperfusion. Nevertheless, 3, 6 and 12 h after ischemic injury all show significant up-regulation of Ero1-α compared to untreated controls (*p* < 0.0001 and 0.01 respectively).

### 3.2. Thapsigargin and Verification of PC12 Cell Suitability

**Figure 2:**
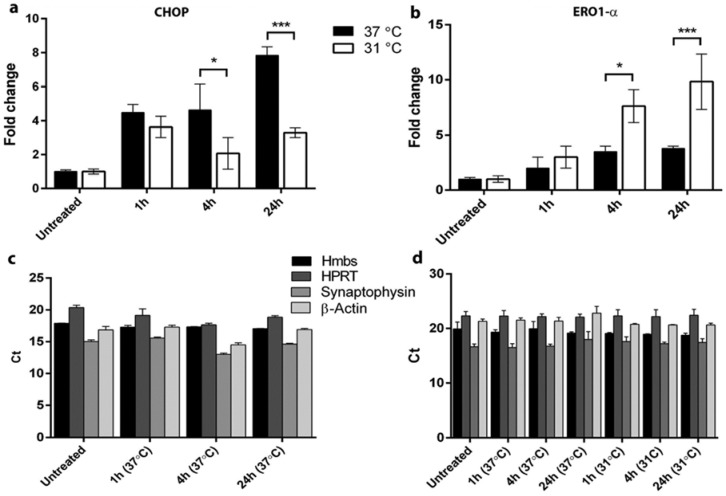
Control experiments performed in order to verify the usefulness of our *in vitro* model and to test the stability of several housekeeping genes *in vitro* and *in vivo*. PC12 cells were incubated under hypoxic conditions for 1 h, 4 h, and 24 h at either 37 °C or 31 °C. Expression levels of CHOP mRNA decreased as a consequence of hypothermia at 4 h (*p* < 0.05) and 24 h (*p* < 0.001) (**a**); whereas ERO1-α mRNA increased following 4 h (*p* < 0.05) and 24 h (*p* < 0.001) (**b**); Threshold cycle values (Ct) of four different housekeeping genes in hippocampus (**c**) and PC12 cells (**d**), revealed that Hmbs was stably expressed under hypoxia at both 31 °C and 37 °C.

Thapsigargin were used in order to verify the suitability of PC12 cells in our experimental setup. Both CHOP (Figure 2a) and ERO1-α (Figure 2b) mRNA were significantly changed following hypothermia, although in opposite directions. Quantification of four different housekeeping genes showed that Hmbs were stably expressed under all experimental conditions (Figure 2c,d).

### 3.3. Hypothermia Attenuates CHOP and Augments Ero1-α Expression in Hypoxic Cells in Vitro

**Figure 3:**
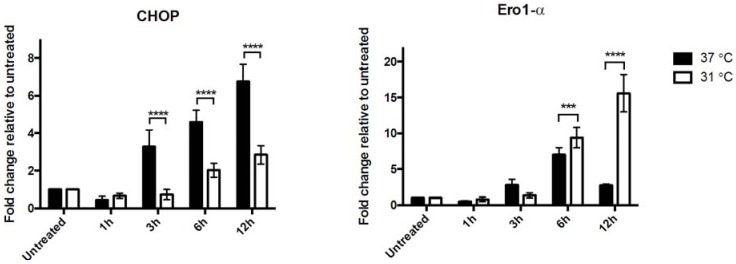
Expression of CHOP and Ero1-α in hypoxic PC12 cells under normo- and hypothermic conditions. Quantitative PCR revealed increased expression levels of CHOP and Ero1-α over a 12 h time course under normothermic conditions. This is consistent with findings in the global ischemia *in vivo* model. Hypothermia attenuated the expression of CHOP significantly (*p* < 0.0001) at 3 (*n* = 6), 6 (*n* = 5) and 12 h (*n* = 5). The expression levels of Ero1-α were augmented by hypothermic conditions with marginally increased levels at 6 h (*n* = 6) and significantly increased levels at 12 h (*n* = 5). *** *p* < 0.001 and **** *p* < 0.0001.

With the use of a SERCA (sarco/endoplasmatic reticulum Ca^2+^-ATPase) pump inhibitor, thapsigargin, a suitable concentration for induction of a UPR response in the PC12 cell line was found (data not shown), confirming the ability of the cell line to activate UPR upon stress. Since we saw a substantial increase in both CHOP and Ero1-α expression levels at 3 h in our *in vivo* model, we included an additional 1 h group subjected to hypothermic conditions. Messenger RNA expression of the two UPR markers was unaffected after 1 h of hypoxia, whereas 3, 6, and 12 h of hypoxia showed similar expression patterns as seen *in vivo* (Figure 3). However, results indicate that PC12 cells respond slightly different to hypoxia compared to hippocampal neurons, where 12 h of hypoxia further increases the expression levels of CHOP, in contrast to the decreased levels observed in our *in vivo* model (Figure 1). Ero1-α expression patterns in PC12 cells also resemble what we found *in vivo*; however, 6 h of hypoxia seemed to entail the greatest increase in expression. Upon incubation at hypothermic conditions, we found a significant decrease in CHOP expression at 3, 6 and 12 h compared to normothermic conditions (*p* < 0.0001). In contrast, expression levels of Ero1-α were increased as a result of hypothermia at 6 h (*p* < 0.001) and 12 h (*p* < 0.0001) (Figure 3).

## 4. Discussion

In our study, global cerebral ischemia was associated with increased transcriptional levels of CHOP and Ero1-α. Whereas high CHOP expression was maintained throughout the study period, ERo1-α peaked at 3 to 6 h (ischemia) and 6 h (hypoxia), respectively. Additionally, we found that hypothermia (31 °C) during hypoxia modulated the ER stress response through downregulation of CHOP after 3, 6 and 12 h and up-regulation of Ero1-α after 6 and 12 h. The fact that these two genes are differentially expressed, both as a consequence of disease induction (hypoxia) and treatment (hypothermia), suggests that they are independently regulated.

Current research suggests that hypothermia causes alterations in the expression of ER stress related genes. Amongst these, an increased expression of the protective GRP78 has been identified, and decreased expression of proapoptotic factor CHOP and, thereby, a reduction in apoptosis [19,20]. Furthermore, hypothermia is known to induce neuroprotection both *in vivo* [19,20] and *in vitro* [21]. Ero1-α has been identified as a CHOP target; however, contradictory evidence on signaling molecules leading to activation of the two isoforms Ero1-α/β exists [15,22,23].

An increase in CHOP expression up to 24 h following global ischemia, associated with death of hippocampal neurons, has previously been shown [19]. Furthermore, 3 h of hypothermia decreased CHOP expression *in vivo* [19] in agreement with our *in vitro* results. 

In contrast, an augmentation of Ero1-α in hypoxic cells at hypothermic conditions was observed. To our knowledge, investigations of the role of Ero1-α in apoptotic pathways following ischemia as well as the influence of hypothermia are lacking. Ero1-α is assumed to be regulated through hypoxia-inducible-factor-1 (HIF-1), a cytosolic transcription factor which is increased in response to low oxygen tension, while Ero1-β is assumed to be regulated through the UPR [22,23]. Furthermore, it has been suggested that Ero1-α is a CHOP target, whilst Ero1-β induction is unaffected by CHOP expression [15]. Since Ero1-α expression in our study is augmented in hypothermic conditions and CHOP is attenuated, a regulation independent of CHOP is indicated. Hence, Ero1-α up-regulation is likely to be attained through the HIF-1 pathway in response to low oxygen tension. Plausibly, hypothermia amplifies the HIF-1 induction, leading to increased expression of Ero1-α in cells stressed with hypoxia.

It should be noted that the PC12 cell line is derived from electrically inexcitable pheochromocytes. Therefore, the results seen in our PC12 cells, in particular the lack of downstream CHOP effects on Ero1-α, should be further confirmed in neurons *in vivo*. Another shortcoming of the study is that our results only address the transcriptional levels. In a broader translational perspective, experiments with the use of transgenic mice relating ischemic damage to specific UPR proteins may highlight whether regulating proteins of the UPR truly is neuroprotective.

In agreement with other studies, we show that an ischemic insult results in significant changes in the transcriptional levels of ER stress regulators [11,24]. Our results draw attention to the potential effect of hypothermia on the UPR. In an *in vivo* and *in vitro* model of ischemic stroke, we provide evidence that CHOP and Ero1-α of the UPR are possible therapeutic targets in ischemic injury. Hypothermia seems to result in a down-regulation of the pro-apoptotic factor CHOP and an increase in Ero1-α expression, thus indicating that hypothermia decouples CHOP-induced transcription of Ero1-α. Our results draw attention to the potential effect of hypothermia on the UPR and future studies of CHOP and Ero1-α regulation at the protein level will further substantiate their feasibility as drug targets in stroke treatment.
